# Membrane protein Nav1.7 contributes to the persistent post-surgical pain regulated by p-p65 in dorsal root ganglion (DRG) of SMIR rats model

**DOI:** 10.1186/s12871-017-0438-8

**Published:** 2017-11-07

**Authors:** Zhisong Li, Yaru Li, Jing Cao, Xuemin Han, Weihua Cai, Weidong Zang, Jitian Xu, Wei Zhang

**Affiliations:** 1 Department of Anesthesiology, the First Affiliated Hospital, Zhengzhou University, No 1, Jianshe Road, Zhengzhou, 450052 People’s Republic of China; 2 Department of Anatomy, Basic Medical College, Zhengzhou University, No 100, Kexue Road, Zhengzhou, 450001 People’s Republic of China; 3 Department of Physiology, Basic Medical College, Zhengzhou University, No 100, Kexue Road, Zhengzhou, 450001 People’s Republic of China

**Keywords:** Persistent post-surgical pain, Mechanical hyperalgesia, DRG, P-p65, Nav1.7

## Abstract

**Background:**

Persistent post-surgical pain is a difficult clinical problem. In this study, we intend to explore the mechanism underlying the persistent post-surgical pain in SMIR (skin/muscle incision and retraction) rats.

**Methods:**

First of all, the expression of membrane protein Nav1.7 and p-p65 (Phosphorylation of p65) were detected in ipsilateral L4–6 DRGs of SMIR rats by western-blot and immunostaining. Then with ProTx-II (Nav1.7 blocker) or PDTC (p65 inhibitor) were intrathecally injected while the change of Nav1.7 expression and mechanical withdrawal threshold were detected. Finally chromatin immunoprecipitation assay method was used to detect whether could p-p65 bind in the Nav1.7 gene promoter region directly.

**Results:**

The results shows that mechanical hyperalgesia occurs following SMIR model, from 5 day (d) and lasted more than 20d after surgery. Meanwhile, the expression of Nav1.7 was up-regulated at 10d, 15d and 20d after surgery compared with naïve group. The expression of p-p65 was up-regulated at 10d and 15d compared with incision group. The mechanical hyperalgesia induced by SMIR was reversed after blocking Nav1.7 or inhibiting p65. Furthermore, Nav1.7 expression was down-regulated when p-p65 was inhibited and p-p65 could combine with the Nav1.7 gene promoter region directly.

**Conclusion:**

Membrane protein Nav1.7 could participate in the peripheral sensitization of persistent post-surgical pain, which may be regulated by p-p65.

## Background

Persistent post-surgical pain (PPP) is a common clinical problem. 4–13% of patients suffer PPP after surgery, including lung cancer, groin hernia repair as well as breast cancer surgery [1]. The PPP severely reduce the quality of patients’ life. Epidemiology shows that many clinical factors could influence the incidence of PPP, including surgical factors, age, sex, preexisting anxiety, depression, increased stress and so on [2, 3]. However, the underlying mechanism of persistent post-surgical pain is still not clear, the relevant molecular biological mechanism need to be explored to support the clinical treatment.

In order to study the peripheral nerve sensitization mechanism of PPP, we need to choose a persistent postoperative pain rat model. Flatters created a typical rat model of persistent postoperative pain by retracting the skin/muscle incision for 1 h without sciatic nerve damage.4 The SMIR model is able to provide a reliable tool for studying PPP. Mechanical hyperalgesia continues for more than 20d in the SMIR model [4–7]. So this model was applied to study peripheral nerve sensitization mechanism of PPP.

Peripheral neurons’ sensitization is the beginning of pain in the signal transduction pathway. The sensitization of peripheral neurons is mostly related to the changes of excitatory transmitter and ion channel receptor expression. Ion channel includes sodium, potassium and calcium ions, among which sodium channel plays a great role in neuropathic pain, especially Nav1.3, Nav1.6, Nav1.7, Nav1.8 and Nav1.9 [8]. In addition, Nav1.7 is one of tetrodotoxin sensitive voltage-gated sodium channel isforms in primarily nociceptive neurons and plays a critical role in regulating neuron excitability [9]. Others studies shows that Nav1.7 plays an essential role in Freund’s Complete Adjuvant (FCA)-induced inflammatory pain as well as STZ-induced diabetic peripheral neuropath [10, 11]. Function deficiency mutations in SCN9A (the gene encodes Nav1.7) can result in a syndrome of congenital inability to experience pain [12]. In our experiment, we found that Nav1.7 was up-regulated at 10d, 15d and 20d after surgery in ipsilateral L4–6 DRGs of SMIR model rats. However, the mechanism of up-regulated Nav1.7 expression is unclear.

NF-κB is an important nuclear transcription factor and composed of different Rel family protein (p65, p50, p52, RelB and c-Rel) [13]. As p65 is one of the most common subtypes localized in the cytoplasm, it may translocate into nucleus to regulate the expression of various genes encoding nociceptive and inflammatory mediators by binding to the promoter region of target genes [14, 15]. We found p-p65 expression was significantly increased in ipisilateral L4–6 DRGs of SMIR. Some studies confirm that the expression of p-p65 is up-regulated in DRG of diabetic peripheral neuropathy model rats and the enhanced interaction between p65 and cbs gene contributes to gastric hypersensitivity in diabetes [16]. However, whether p65 could regulate Nav1.7 in the development of the PPP-induced allodynia is unknown.

## Methods

### Animals, drugs and drug administration

Male Sprague-Dawley rats weighing 220–250 g were hosted at a constant ambient temperature of 23 ± 2 °C under a 12:12-h light-dark cycle. All the experimental animals were hosted in a single cage respectively with free access to water and food. The entire experimental program is approved by Zhengzhou University Animal Care and Use Committee and in accordance with animal ethical standards [17]. Briefly, ProTx-II (Tocris, No.4023) dissolved in 0.9% normal saline (0.1 mg/m L) was intrathecal (i.t) injection (8 μ g/kg, 4 μ g/kg, 2 μ g/kg) once in a day for 8 consecutive days from the day before operation; PDTC (Sigma, P8765) dissolved in 0.9% normal saline (0.05 mg/m L) was i.t injection (0.2 μ g, 0.5 μ g) once in a day for 8 consecutive days from the day before operation.

### Persistent postoperative pain model

Rats (220–250 g) were anaesthetized with intra-peritoneal (i.p) injection of 10% chloral hydrate at doses of 300 mg/kg. An incision (1.5–2 cm) was made on the skin of the medial thigh about 4 mm medial to the saphenous vein, and then a 7–10 mm incision was made on the superficial muscle layer. A retractor (NO.R22009–02, RWD Life Science Inc.) was inserted into the incision site. The incision of the thigh was retracted to 2 cm, while the process of retraction last for 1 h. Then the surgical site was covered by sterile gauze in order to prevent loss of body fluids and heat. In the end, the skin and muscle incision were closed with medical silk. The whole procedure was made according to previous research [4].

### Intrathecal catheter implantation

In the preoperative day, PE-10 catheter was steeped in medical alcohol overnight and flushed by normal saline in the next day. Rats (200–220 g) were anaesthetized with i.p (intraperitoneal injection) injection of 10% chloral hydrate. The whole procedures were performed as described by Storkson etc [18]. Briefly, after skin preparation, about 2 cm incision was made in the center of backside from L5 to S1. Spinal dura mater was punctured by a needle after being exposed the intravertebral space between L5 and L6, then the catheter was inserted into subarachnoid space to reach the lumbar enlargement. Medical silk was used to fix PE-10 catheter, and incision was closed layer by layer. 10 u L lidocaine (2%) was i.t injected to verify whether the catheter was successfully inserted. There are 7 days times for rats to recover. Animals were excluded from experiments if they showed any signs of nerve damage.

### Behavioral test

Mechanical sensitivity was assessed by the up-down method and the paw-withdrawal threshold (PWT) with Von Frey hairs [19]. Before the behavior test, experimental animals were placed in a cage for 1 h to adapt to the environment. The hind paw of rats was stimulated by Von Frey hairs, each stimulus last for 2 s, and the interval was 5 min. Rapid withdrawal or licking of the paw in response to the stimulus was regarded as a positive response. All behavioral tests were performed by a researcher blinded to the experimental group.

### Western blotting

All the samples in the experiment were stored at −80 °C. Three or four rats were randomly assigned to each group. The ipsilateral L4-L6 DRGs were homogenized using a glass homogenizer in chilled lysis buffer ‘A’ containing sucrose, 1 M Tris pH 7.4, 1 M MgCl_2_, 0.25 M EGTA, 0.5 M PMSF, 100 m M DTT, 2 m M leupeptin, NP-40, cocktail and phosphatase inhibitors. After centrifugation for 15 min at 4 °C and 1000 g, the supernatant was collected for cytosolic protein, then the precipitation was homogenized using Ultrasonic Cell Disruptor in buffer ‘B’ containing 1 mM Tris, 20% SDS, tritonX 100, cocktail and phosphatase inhibitors. After centrifugation for 15 min at 4 °C and 12,000 g, the supernatant was collected for nucleoprotein. Protein concentration was detected by the Bradford method (Solarbo BCA Protein Assay Kit, PC0010). Equal amount of protein was separated by PAGE (25 u g per line) and transferred onto PVDF membranes, which were blocked with 5% BSA for 2 h and incubated with primary antibody over two nights at 4 °C. After that the membranes were incubated with general secondary antibody. The above antibodies include rabbit anti-Nav1.7 (Merck Millipore, AB5390, 1:1000), rabbit anti-phospho-p65-Ser536 (Cell Signaling Technology, 3033, 1:1000), rabbit anti-H3 (Sangon Biotech, E021110–01, 1:1000) and mouse anti-β-actin (Sigma, A1978, 1:10,000). The immune complex was exposed with ECL and the signal intensity was analyzed by using Alphaview SA software.

### Immunohistofluorescence

Rats (220–250 g) were anesthetized with i.p. 10% chloral hydrate at dose of 300 mg/kg, and perfused with 250 m L normal saline and 250 m L 4% ice-cold paraformalde (PFA) through the ascending aorta. The ipsilateral L4-L6 DRGs were collected, then post-fixed in 4% PFA overnight and dehydrated in 30% sucrose in PBS solution at 4 °C until it sank to the bottom. The DRG was sliced to longitudinal sections (16 μ m) and then were washed three times with PBS solution. They were blocked with 5% BSA for 2 h at room temperature and incubated with primary antibodies overnight at 4 °C, following by being washed three times with PBS solution and incubated with secondary antibodies. For double immunohistofluorescence staining, the primary antibodies and secondary antibodies were mixed respectively. Primary antibodies used in previous experiments included rabbit anti-Nav1.7 (1:100, Merck Millipore, AB5390), rabbit anti-phospho-p65-Ser536 (1:100, Cell Signaling Technology, 3033), mouse anti-GFAP (1:200, abcam, ab10062), mouse anti-CGRP (1:200, abcam, ab81887), mouse anti-NF-200 (1:100, Boster, M05307–1) and IB-4(1:100, Sigma, L2895). The stained sections were posted on glass slides with a very fine brush and the images were captured through image J software by fluorescence microscope (Nikon TE 2000-E, Melville, NY).

### ChIP (chromatin immunoprecipitation) assay

The ChIP assays were performed using the Pierce Agarose ChIP Kit (Thermo Scientific, 26,156). Primers of Nav1.7 proximal promoter region designed by Sangon Biotech (Table 1).The ipsilateral L4-L6 DRGs neurons cell of SMIR rats were isolated with ice-cold PBS (1X) containing 1% cocktail. The isolated DRG neurons cell was crosslinked with 1% formaldehyde for 10 min in a chemical fume hood at room temperature and then was terminated by glycine solution (1X). The crosslinked cells prepared above were lysed and digested until the DNA fragments was broken into 200-1000 bp. After the DNA fragments were pre-cleaned with ChIP grade protein A/G plus agarose, they were respectively subjected to immunoprecipitation over night at 4 °C on a rocking platform with rabbit antibody against p-p65 and normal rabbit IgG. Input (10% sample for immunoprecipitation) was used as a positive control. The DNA fragments were identified using PCR with primers.

**Table 1:** Primer sequences for SCN9A proximal promoter region

Target gene	No.	Primers	Primer sequence
SCN9A	1	Forward	5′-ATGAAATGGTGCTGCCTACA-3’
		Reverse	5′-CCTGGGTGGAAGTGAAGAAA-3’
	2	Forward	5′-ATGGTGCTGCCTACATTCAAG-3’
		Reverse	5′-TGGGTGGAAGTGAAGAAAGG-3’
	3	Forward	5′-CAACACACACACACACATGGA-3’
		Reverse	5′-TGCACAAGGGCTTTTACTCTG-3’
	4	Forward	5′-GAGTCCCAGGCCATGAAAT-3’
		Reverse	5′-TATCCAGTCTTTGGGGATGC-3’
	5	Forward	5′-GGGTGTTGTAGGTCACATTGG-3’
		Reverse	5′-ATGAGGGCAAGGGACTGTTT-3’
	6	Forward	5′-TGAAATGGTGCTGCCTACAT-3’
		Reverse	5′-TGGGTGGAAGTGAAGAAAGG-3’
	7	Forward	5′-AATGGTGCTGCCTACATTCA-3’
		Reverse	5′-CCTGGGTGGAAGTGAAGAAA-3’
	8	Forward	5′-GGGTGTTGTAGGTCACATTGG-3’
		Reverse	5′-AGGGCAAGGGACTGTTTTGT-3’
	9	Forward	5′-TGGTGCTGCCTACATTCAAG-3’
		Reverse	5′-TGGGTGGAAGTGAAGAAAGG-3’

### Statistical analysis

Data were represented as mean ± SEM. Statistical analysis was performed using Prism 5.0 software. One-way ANOVA for western blotting data and Two-way ANOVA for behavior data, *P* < 0.05 would be considered significant.

## Results

### The SMIR model rats mediated mechanical hyperalgesia of ipsilateral hind paw plantar

**Fig. 1:**
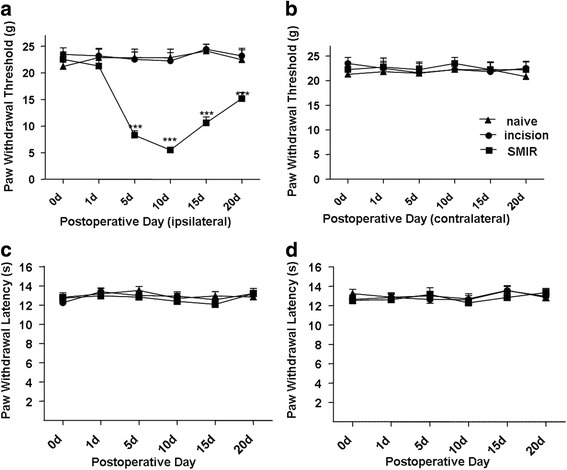
**a**-**d** The PWT result showed that persistent postoperative pain can be evoked through prolonged tissue retraction, consistent with the previously reported. Incision group (incision), SMIR group (incision plus retraction). (**P < 0.01, ***P < 0.001, vs incision group, *n* = 8)

Between incision group (incision but no retraction) and naive group, PWT (paw withdrawal threshold) were no statistical difference. Ipsilateral PWT in SMIR group was significantly decreased from 5d after the SMIR surgery to 20d compared with the incision group, while no significant difference exists in contralateral PWT between incision group and SMIR group. Compared with naive group, paw withdrawal latencies of bilateral hind paw plantar in SMIR group rats were not significantly changed, indicating that SMIR model was successfully established (Fig. 1a-d).

### Up-regulation of Nav1.7 in DRG of SMIR rats was involved in the hyperalgesia

**Fig. 2:**
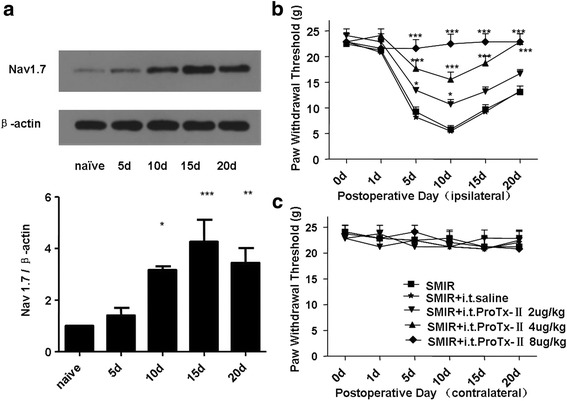
**a** Western blotting of Nav1.7 using the total protein from ipsilateral L4-L6 DRGs of SMIR surgery rats, statistical summaries of western blot analysis: The expression of Nav1.7 protein was increased on days 10,15 and 20 after SMIR surgery (*P < 0.05,**P < 0.01, ***P < 0.001 vs naive group; *n* = 3). **b**-**c** Effect of pretreatment with i.t injection of ProTx-II (once daily from 1d before surgery until 7d after surgery) on the mechanical hyperalgesia induced by SMIR model rats. The result showed that selective Nav1.7 channel blocker can significantly reduced hyperalgesia induced by SMIR in a dose dependent way (*P < 0.05, ***P < 0.001 vs SMIR group; *n* = 6)

We performed western blotting analysis, which revealed that Nav1.7 expression in the ipsilateral DRG of the SMIR rats was significantly up-regulated on 10d and last until 20d (Fig. 2a). To ascertain whether the up-regulated Nav1.7 plays a very important role in the mechanical hyperalgesia of SMIR rats, behavior change of SMIR rats were detected by i.t injection of ProTx-II. ProTx-II was found to effectively alleviate mechanical hyperalgesia of ipsilateral paws induced by SMIR, but had no effect on contralateral paws (Fig. 2b-c).

### Nav1.7 was expressed in positive cells of CGRP, NF-200 and IB-4

**Fig. 3:**
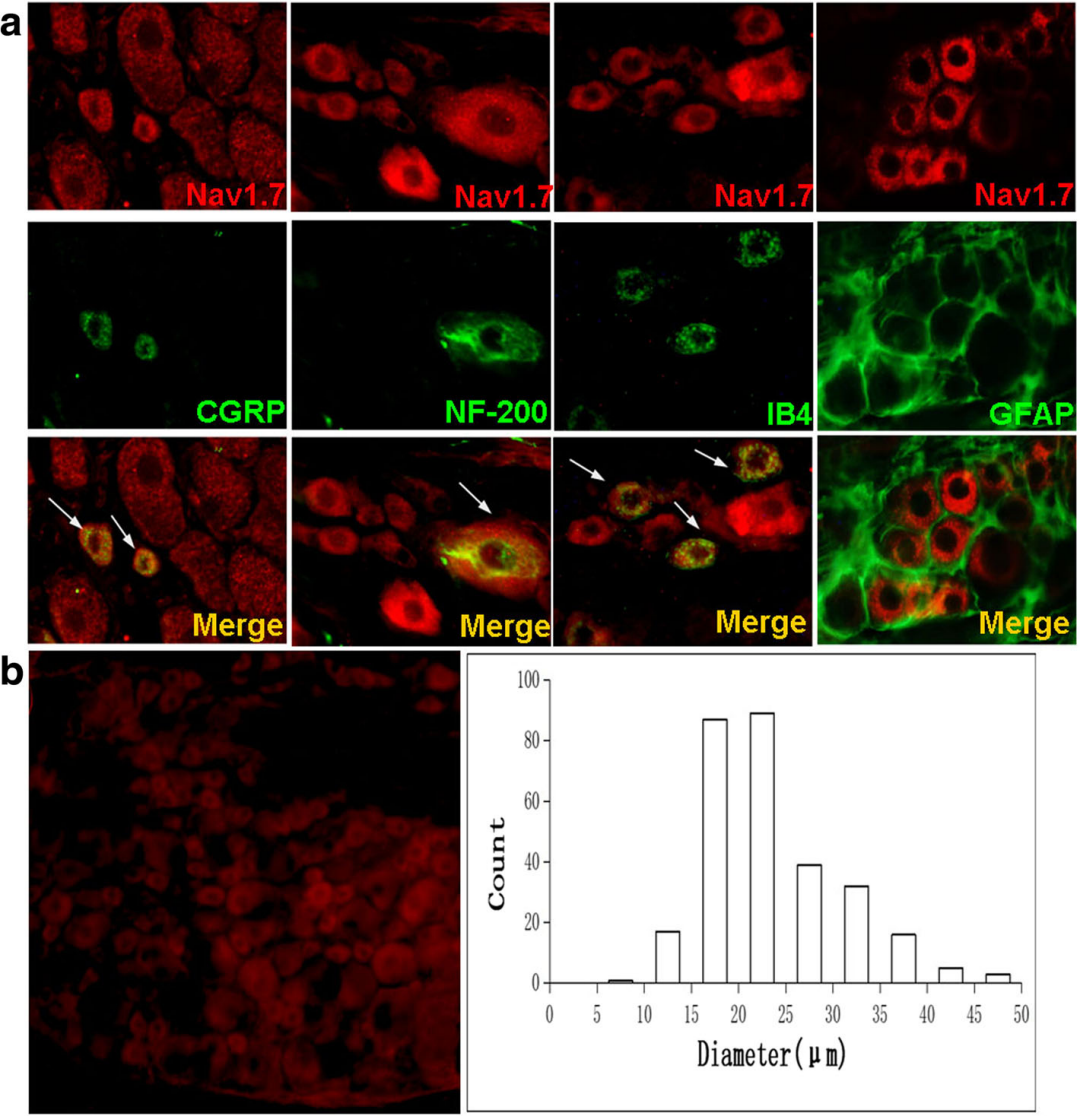
**a** Double immunofluorescence staining (The ipsilateral DRGs after 10d of SMIR group rats were used). **b** The distribution of Nav1.7 positive cells diameter (DRG from 3 rats)

To further determine the cell-types of Nav1.7 expression in the DRG neurons, double immunofluorescence staining method was used. The result showed that Nav1.7 was expressed in positive cells of CGRP, NF-200, IB-4 but not in cells of GFAP (a specific marker of astrocyte) (Fig. 3a). Cell diameter was measured by ImageJ software, we found that about 80% Nav1.7 positive cells are small diameter neurons, 17% are medium diameter neurons, and only 3% are large-diameter neurons (Fig. 3b).

### P-p65 was over expressed in DRG of SMIR rats

**Fig. 4:**
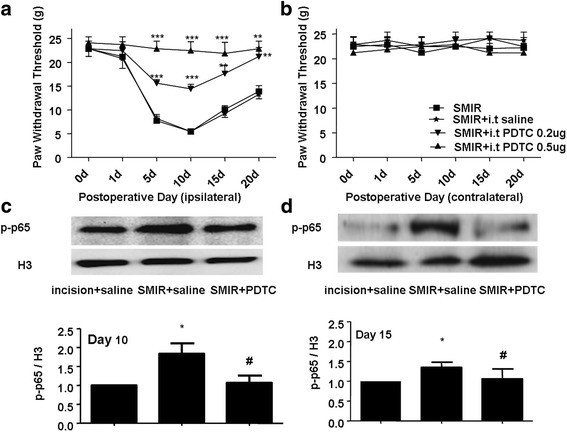
**a**-**b** Effects of pretreatment with i.t injection of PDTC (once daily from 1d before surgery until 10d after surgery) on the mechanical hyperalgesia induced by SMIR model (**P < 0.01,***P < 0.001,*n* = 6). **c**-**d** Western blotting result of nucleoprotein p-p65 from ipsilateral L4-L6 DRGs of SMIR surgery rats. Statistical summaries of western blot analysis: The expression of p-p65 protein was increased at 10d (*n* = 4) and 15d (*n* = 5) after SMIR surgery compared with incision groups, whereas pretreatment with PDTC (once daily from 1d before surgery until 7d after surgery) can prevent the activation of NF-κB at 10d (*n* = 4) and 15d (*n* = 5) compared with SMIR group (*P < 0.05 vs incision group; #*P* < 0.05 vs SMIR group)

As it has been reported, p-p65 (phosphorylated NF-κB) involved in process of neuropathic pain and inflammatory pain. In the study, the role p-p65 played in persistent post-surgical pain was detected. PDTC, an inhibitor of NF-κB, was found to prevent ipsilateral paw hyperalgesia induced by SMIR rats, but had no effect on contralateral paw (Fig. 4a-b). Western blotting result showed nucleoprotein p-p65 in rat ipsilateral L4-L6 DRGs at 10d and 15d after SMIR surgery increased obviously comparing with incision group; however the level of p-p65 in group pretreated with PDTC 0.5μg was down-regulated comparing with SMIR group (Fig. 4c-d).

### P-p65 contributed to the persistent post-surgical pain through up-regulated expression of Nav1.7 in DRG of SMIR rats

**Fig. 5:**
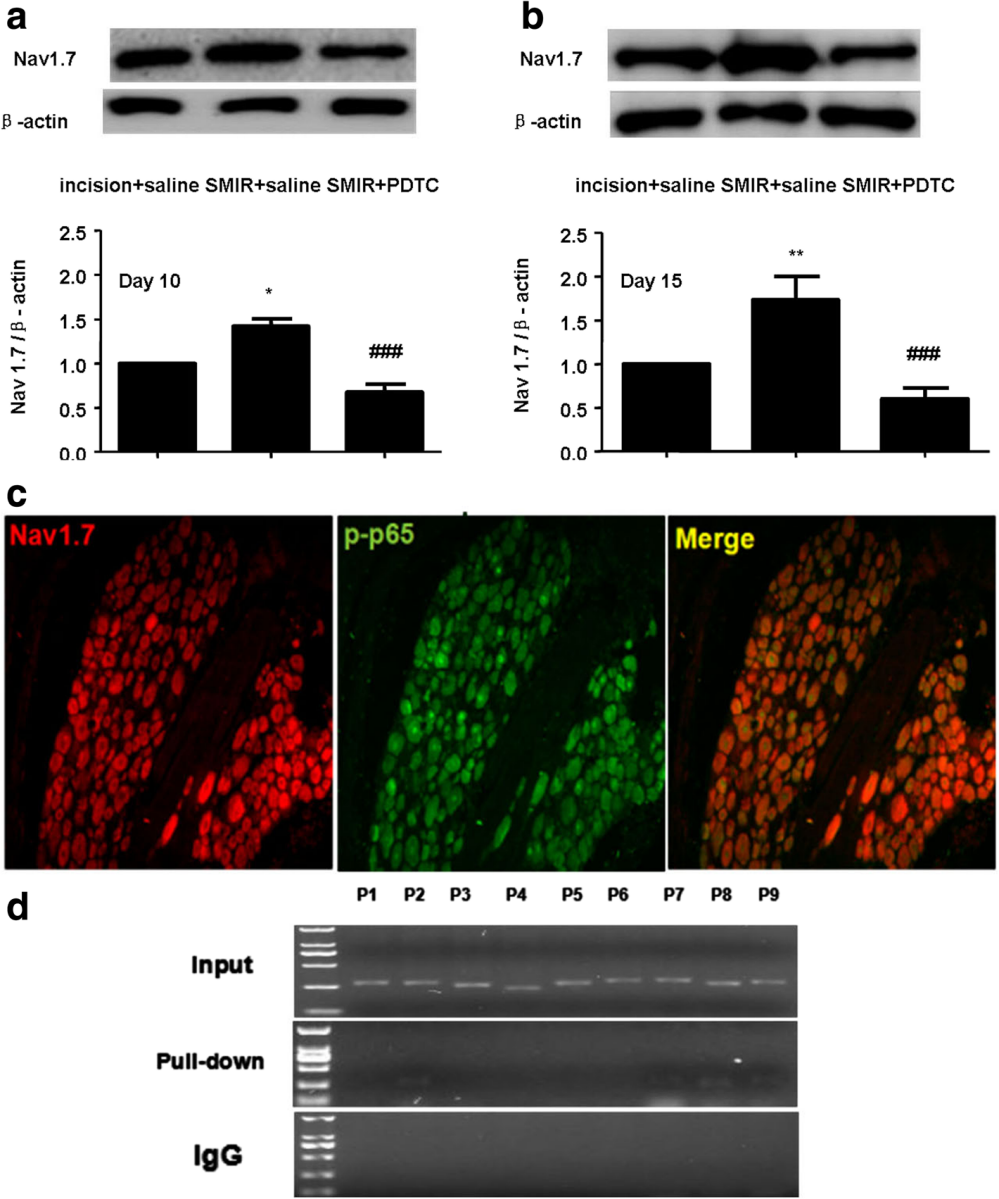
**a**-**b** Western blotting result of Nav1.7 from ipsilateral L4–6 DRGs of SMIR rats. Statistical summary of the densitometric analysis: compared to SMIR group, pretreatment with PDTC can inhibit the up-regulation of Nav1.7 at 10d and 15d after surgery (*P < 0.05,**P < 0.01 vs incision group; ###*P* < 0.001 vs SMIR group, *n* = 3). **c** Double immunofluorescence staining of ipsilateral DRG (10d after SMIR surgery). **d** Products of PCR amplifications for P1 - P9 from the SCN9A proximal promoter region was separated by 1% agarose gel electrophoresis

As it is known, p-p65 is a significant transcription factor that regulates transcription of multiple target genes. To further investigate whether p-p65 participates in the regulation of Nav1.7 in the persistent post-surgical pain model, PDTC was i.t injected to detect the expression change of Nav1.7. The result indicated that compared with incision group, Nav1.7 was increased and PDTC could prevent the up-regulation of Nav1.7 induced by SMIR at 10d and 15d after surgery (Fig. 5a-b). Next double immunohistofluorescence stain was done. p-p65 was found to have co-localized with Nav1.7 (Fig. 5c). ChIP assay indicated p-p65 could combine with SCN9A proximal promoter region for primer 2, primer 7, primer 8, primer 9 (Fig. 5d).

## Discussion

PPP becomes a serious clinical problem and severely impacting the quality of patients’ life. We established the SMIR model in rats and successfully elicit the mechanical hyperalgesia. Compared with naive group, the expression of Nav1.7 was strongly increased at 10d, 15d and 20d after surgery in ipsilateral L4–6 DRGs of SMIR rats. Blocking Nav1.7 could reserve the mechanical hyperalgesia caused by operation. Meanwhile, p-p65 expression was up-regulated at 10d and 15d after surgery in ipsilateral L4–6 DRGs of SMIR rats. Intrathecal injection of PDTC could reverse mechanical hyperalgesia, concurrent with down- regulated Nav1.7 expression at 10d and 15d after surgery. P-p65 could bind with SCN9A proximal promoter region for primer 2, primer 7, primer 8, primer 9. Above all, we found Nav1.7 could participate in the peripheral sensitization of persistent post-surgical pain via regulated by p-p65.

Epidemiology shows acute pain disappear 5–7 days after operation in the majority of patients, however, 5–10% patients still suffer pain more than 1 month after wound healed, which is called PPP. It brings huge burden to patients and society. It is difficult for clinical treatment because the mechanism is unclear. In order to study the mechanism of PPP, SMIR rat model was applied [4]. In addition, the procedure of SMIR model is akin to clinical operates. The results showed that SMIR evoked mechanical hypersensitivity in the ipsilateral hind paw but not heat hyperalgesia (Fig. 1a-d), but there weren’t any changes on contralateral and incision group.

It is widely known that peripheral sensitization was involved in development and maintains of pain, many researches demonstrated that peripheral sensitization correlates with ion channel receptors. For example, a study reported that over-expressed voltage-gated potassium channel subunit Kv1.2 could alleviate neuropathic pain [20]. Another research revealed that blocking N-type (Ca (V)2.2) calcium channels can treat intractable pain [21]. Voltage-gated sodium channels Nav1.7 and Nav1.9 were reported participate in neuropathic pain and inflammatory pain [22, 23]. So we focus on the effect of Nav1.7. In this study, we detected the change of Nav1.7 expression in SMIR model. Results showed Nav1.7 expression has increased in ipsilateral L4–6 DRGs of SMIR rats at 10d, 15d and 20d after surgery (Fig. 2a). ProTx II could reverse paw mechanical hyperalgesia (Fig. 2b-c). It illustrated that Nav1.7 involved in the process of PPP. Nav1.7 is predominantly expressed in peripheral nervous system [24], so it may have little side effects. These results consist with others previous studies that Nav1.7 function enhanced mutation contributed to idiopathic small fiber neuropathy and Nav1.7 protein was high-expressed in DRG neurons of rats with diabetic neuropathy [25, 26].

In our study, we found p-p65 expression was increased in the SMIR model at 10d and 15d after surgery (Fig. 4c-d). Other studies have reported that Nav1.7 could be regulated by PKA, PKC and MAPK pathway [11, 27, 28], as a crucial nuclear transcription factor, p65 participates in a variety of organism pathophysiological processes, no research has yet found that p-p65 regulates Nav1.7 in PPP directly.

Previous studies have linked enhanced interaction between p-p65 and cbs gene with gastric hypersensitivity in diabetes [16]. Our results showed that SMIR increased expression of p-p65 (Fig. 4c-d) and PDTC prevented the up-regulation of Nav1.7 (Fig. 5a-b) while alleviated the hyperalgesia induced by SMIR (Fig. 4a-b). P-p65 could combine with SCN9A proximal promoter region (Fig. 5d). These findings suggested that voltage-gated sodium channel Nav1.7 expression play a crucial role in PPP, which could be up-regulated by p-p65 in L4–6 DRGs of SMIR model rats. Based on these results, Nav1.7 may become the target of analgesic drugs.

## Conclusion

In summary, this study demonstrated that p-p65 induced up-regulated voltage-gated sodium channel Nav1.7 contributed to the PPP.
